# Sex‐ and *APOE*‐specific genetic risk factors for late‐onset Alzheimer's disease: Evidence from gene–gene interaction of longevity‐related loci

**DOI:** 10.1111/acel.13938

**Published:** 2023-08-24

**Authors:** Serena Dato, Francesco De Rango, Paolina Crocco, Stefano Pallotti, Michael E. Belloy, Yann Le Guen, Michael D. Greicius, Giuseppe Passarino, Giuseppina Rose, Valerio Napolioni

**Affiliations:** ^1^ Department of Biology, Ecology and Earth Sciences University of Calabria Rende Italy; ^2^ Genomic And Molecular Epidemiology (GAME) Lab., School of Biosciences and Veterinary Medicine University of Camerino Camerino Italy; ^3^ Department of Neurology and Neurological Sciences, School of Medicine Stanford University Stanford California USA

**Keywords:** Alzheimer's Disease, epistasis, gene–gene interaction, IGF1, longevity, polymorphism

## Abstract

Advanced age is the largest risk factor for late‐onset Alzheimer's disease (LOAD), a disease in which susceptibility correlates to almost all hallmarks of aging. Shared genetic signatures between LOAD and longevity were frequently hypothesized, likely characterized by distinctive epistatic and pleiotropic interactions. Here, we applied a multidimensional reduction approach to detect gene–gene interactions affecting LOAD in a large dataset of genomic variants harbored by genes in the insulin/IGF1 signaling, DNA repair, and oxidative stress pathways, previously investigated in human longevity. The dataset was generated from a collection of publicly available Genome Wide Association Studies, comprising a total of 2,469 gene variants genotyped in 20,766 subjects of Northwestern European ancestry (11,038 LOAD cases and 9,728 controls). The stratified analysis according to APOE*4 status and sex corroborated evidence that pathways leading to longevity also contribute to LOAD. Among the significantly interacting genes, *PTPN1*, *TXNRD1*, and *IGF1R* were already found enriched in gene–gene interactions affecting survival to old age. Furthermore, interacting variants associated with LOAD in a sex‐ and *APOE*‐specific way. Indeed, while in APOE*4 female carriers we found several inter‐pathway interactions, no significant epistasis was found in APOE*4 negative females; conversely, in males, significant intra‐ and inter‐pathways epistasis emerged according to APOE*4 status. These findings suggest that interactions of risk factors may drive different trajectories of cognitive aging. Beyond helping to disentangle the genetic architecture of LOAD, such knowledge may improve precision in predicting the risk of dementia and enable effective sex‐ and *APOE*‐stratified preventive and therapeutic interventions for LOAD.

## Graphical abstract

Genetic studies over the last few years, have changed the perception of late‐onset Alzheimer's disease (LOAD), highlighting its polygenic nature with multiple susceptibility genetic loci. We applied a multidimensional reduction approach to detect gene–gene interactions affecting LOAD by testing variants harbored by genes previously investigated in human longevity. This study suggests that longevity loci may also drive LOAD neuropathology through *APOE*‐ and sex‐related specific gene–gene interactions.

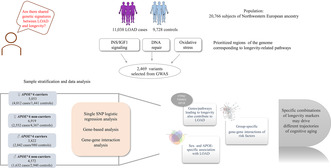

**Abbreviations**
AD: alzheimer's disease
APOE: apolipoprotein E
CV: cross‐validation
GWAS: genome wide association study
IBD: identity‐by‐descent
IG: information gain
IGF1: insulin growth factor 1
LD: linkage disequilibrium
LOAD: late‐onset alzheimer's disease
MAF: minor allele frequency
MDR: multifactor dimensionality reduction
QTL: quantitative trait locus
SNP: single nucleotide polymorphism

## 1 INTRODUCTION

Alzheimer's disease (AD) is the most prevalent disease among people over 85 years of age in western countries, posing a significant challenge to public health systems around the world. Most AD cases are sporadic or late onset (LOAD; >65 years of age), with biological measures of disease being detectable as early as 20 years before the first cognitive symptoms are observed (Jagust, 2018). Decades of research have shed light on the neuropathological changes happening in the AD brain, and its complex etiology (Long & Holtzman, 2019), characterized by sex differences in several aspects of the disease, including its onset and progression, and the effects of APOE*4 genotype, the strongest common genetic risk factor for LOAD (Nebel et al., 2018). A current challenge is to clarify the contribution of genetic, epigenetic, and environmental factors in the multifactorial nature of LOAD, which shows a heritability of 58%–79%, with a large fraction attributable to the *APOE* locus. Genetic studies over the last few years, particularly coming from genome‐wide association studies (GWAS) and large sequencing projects, have changed the perception of LOAD, highlighting its polygenic nature with multiple susceptibility genetic loci (Andrews et al., 2023). Also, functional genomic analyses pointed out that common LOAD risk variants operate in complex networks of genetic and metabolic interactions, regulated by “hub” genes and “peripheral master regulators” (Gui et al., 2021). In the interactome network of the cell, each variant may show different effects (either in magnitude or in direction) on disease onset, in relation to alleles at other loci (Ridge et al., 2016). These epistatic effects may contribute substantially to the variation in disease susceptibility; that is, people carrying risk factors for LOAD but resilient to the disease, as well as people carrying the risk allele APOE*4 who live into their 90s without developing dementia.

While advances in technologies and increased availability of multi‐omics data have expanded our knowledge about LOAD genetic architecture, many risk variants remain to be identified (Andrews et al., 2023). Such variants may be found in the underlying processes leading to LOAD, such as inflammation (Akiyama et al., 2000), apoptosis (Behl, 2000), stress response (Iatrou et al., 2021), and mitochondrial decay (Kwong et al., 2006). Interestingly, many of these mechanisms are common to human longevity, suggesting that the search of susceptibility factors could be enhanced by testing genes belonging to pathways influencing longevity and survival. As a proof‐of‐concept alongside *APOE*, which is the locus consistently associated with longevity across different populations (Abondio et al., 2019), several other LOAD‐associated loci were found to affect the human lifespan (Tesi et al., 2021). Moreover, several studies reported LOAD risk variants that are associated with both AD and longevity (Bacalini et al., 2022; Dato et al., 2021; Tesi et al., 2021), emphasizing the importance of studying the pleiotropic effects and epistatic interactions of genetic variants.

Thus, searching for epistatic interactions between variants in genomic regions selected for their association with longevity may help to unravel some of the missing genetic variance of LOAD. To test this hypothesis, we leveraged a large collection of publicly available LOAD GWAS data and conducted gene–gene interaction analysis to find meaningful associations. Genomic regions were prioritized based on previous work by Dato and coworkers (Dato et al., 2018), who analyzed the joint effect on longevity of SNPs belonging to three candidate pathways, the insulin/insulin‐like growth factor signaling (IIS), DNA repair, and stress response, respectively. These pathways were chosen as they regulate fundamental biological processes consistently recognized among the most relevant hallmarks of aging from model organisms to humans (Kuningas et al., 2008) and confirmed to have an important role in human longevity in large cohort studies (Deelen et al., 2019).

The analyzed dataset comprised genotypes for a total of 2469 gene variants from 20,766 subjects (11,038 LOAD cases and 9728 controls), belonging to several LOAD cohorts of Northwestern European ancestry. As we stated above growing evidence indicates sex‐specific patterns of disease manifestation and sex‐dependent effects of *APOE* on LOAD risk. Yet, beyond APOE*4, other genetic risk factors have been found that display sex‐specific effects on LOAD, and the interplay between sex and the *APOE* allele has been also explored (Fan et al., 2020). In addition, sex‐specific DNA methylation changes in LOAD pathology have been observed (Zhang et al., 2021). On the other hand, also the sex differences in genetic associations with longevity are remarkable (Zeng et al., 2018). Based on this evidence, we reasoned that stratifying genetic association analysis by sex and *APOE* status may facilitate the identification of sex‐specific genetic risk loci and ultimately contribute to the understanding of disease heterogeneity between men and women.

## 2 MATERIALS AND METHODS

### 2.1 Demographics & study datasets

Fifteen late‐Onset Alzheimer's Disease (LOAD) GWAS datasets were obtained from publicly available data repositories (Table 1). Genotyping was performed using various high‐density single‐nucleotide variant microarrays across cohorts. Participants or their caregivers provided written informed consent in the original studies. The current study protocol was granted an exemption by the Stanford University institutional review board because the analyses were carried out on deidentified, off‐the‐shelf data.

**TABLE 1:** Demographic information for individuals in the analysis dataset.

		LOAD		Controls		Mean age LOAD (SD)		Mean age controls (SD)		APOE*4 LOAD		APOE*4 controls	
Cohort	N	F	M	F	M	F	M	F	M	F	M	F	M
ACT	2,115	343	190	872	710	81.7 (±6.4)	80.4 (±6.4)	82.5 (±6.1)	81.7 (±5.9)	149 (43.4%)	91 (47.9%)	201 (23.1%)	137 (19.3%)
ADCC	6,427	2,171	1,800	1,561	895	74.1 (±8.0)	72.5 (±7.2)	79.5 (±9.0)	80.1 (±8.6)	1,411 (65.0%)	1,201 (66.7%)	389 (24.9%)	233 (26.3%)
ADDNEUROMED	239	77	47	64	51	73.7 (±7.7)	74.2 (±6)	78.5 (±6.6)	78.5 (±5.8)	48 (62.3%)	28 (59.6%)	17 (26.6%)	15 (29.4%)
ADNI	749	184	247	163	155	74.3 (±6.6)	75.8 (±6.6)	78.7 (±6.4)	79.6 (±7.4)	128 (69.6%)	168 (68.0%)	46 (28.2%)	42 (27.1%)
GenADA	1,375	400	286	436	253	74.0 (±7.3)	73.9 (±6.2)	73.9 (±7.2)	75.0 (±6.9)	257 (64.3%)	184 (64.3%)	100 (22.9%)	65 (25.7%)
HBTRC	302	123	100	22	57	73.9 (±5.5)	70.3 (±6.1)	70.3 (±6.9)	68.9 (±6.8)	67 (54.5%)	52 (52.0%)	5 (22.7%)	23 (40.4%)
NIA‐LOAD	1,544	559	311	421	253	72.7 (±6.8)	71.9 (±6.7)	75.3 (±9.4)	75.8 (±8.6)	414 (74.1%)	236 (75.9%)	133 (31.6%)	83 (32.8%)
MAYO	1,730	432	292	533	473	73.8 (±5.0)	73.9 (±5.0)	73.7 (±4.5)	73.4 (±4.3)	286 (66.2%)	192 (65.8%)	153 (28.7%)	134 (28.3%)
MIRAGE	466	159	97	117	93	70.6 (±6.4)	70.8 (±7.0)	71.5 (±7.6)	72.2 (±7.9)	106 (66.7%)	59 (60.8%)	42 (35.9%)	37 (39.8%)
OHSU	379	114	64	108	93	86.9 (±6.3)	84.6 (±5.1)	87.7 (±7.4)	86.5 (±7.3)	39 (34.2%)	31 (48.4%)	23 (21.3%)	24 (25.8%)
ROSMAP	1,131	468	180	356	127	84.3 (±6.6)	83.8 (±6.5)	85.8 (±7.1)	86.2 (±6.8)	160 (34.2%)	65 (36.1%)	74 (20.8%)	18 (14.2%)
TGEN	1,012	415	205	188	204	74.0 (±6.7)	72.2 (±6.6)	82.5 (±8.8)	79.3 (±8.5)	277 (66.8%)	132 (64.4%)	39 (20.7%)	43 (21.1%)
UPITT	1,667	627	356	437	247	73.2 (±6.8)	73.0 (±6.5)	75.9 (±6.3)	75.2 (±5.9)	365 (58.2%)	214 (60.1%)	84 (19.2%)	51 (20.7%)
UVM	1,257	354	193	444	266	73.6 (±7.7)	71.5 (±7.3)	73.4 (±7.6)	74.2 (±7.4)	225 (63.6%)	134 (69.4%)	111 (25.0%)	63 (23.7%)
WASHU	373	138	106	86	43	76.6 (±9.5)	75.3 (±8.3)	77.6 (±9.9)	76.5 (±6.6)	80 (58.0%)	55 (51.9%)	24 (27.9%)	12 (27.9%)
Total	20,766	6,564	4,474	5,808	3,920								

Abbreviations: F, Females; M, Males.

### 2.2 Genotyping data: Harmonization & imputation

The entire dataset includes 35,110 participants. Adopted SNP‐array Quality Control (QC) procedures were like the ones previously reported (Napolioni et al., 2021). Subjects with autosomal missingness >5% and/or X‐chromosome missingness >5% (compared to other subjects in the same dataset), age below 60 years, age information missing, or phenotype inconsistency [missing phenotype, diagnosis of mild cognitive impairment, or a neurodegenerative phenotype other than LOAD] were excluded from the analysis.

Individual ancestry was determined using SNPweights v.2.1 (Chen et al., 2013) by using reference populations from the 1000 Genomes Consortium (1000 Genomes Project Consortium, 2015). By applying an ancestry percentage cut‐off > = 80%, the samples were stratified into five super populations, South‐Asians (SAS), East‐Asians (EAS), Americans (AMR), Africans (AFR), and Europeans (EUR). Since most of the samples belonged to the European population, we also determined their ancestry percentage in the discovery sample according to three genetically distinct European sub‐ancestries, Northwestern, Southeastern, and Ashkenazi Jewish, using reference populations available from SNPweights v.2.1. European subjects were stratified into the above‐mentioned ancestries when their ancestry percentage was > = 50% for any of the three sub‐ancestries. Subjects with genetic ancestry estimates discordant from self‐reported ancestry, as well as for subjects showing sex‐inconsistency, were excluded from the analyses.

After keeping only the EUR subjects, each GWAS dataset was QCed to remove the SNPs with a call rate ≤95%; Minor Allele Frequency (MAF) ≤1%; SNPs with MAF deviating more than 10% from the MAF reported in 1000 Genomes for the EUR population; SNPs with differential missingness between cases and controls (*p* < 0.05); SNPs deviating from Hardy–Weinberg Equilibrium (HWE) in controls (*p* < 5 × 10^−5^); tri‐allelic SNPs; and SNPs where the alleles are mismatched compared to the 1000 Genomes reference sequence. A/T and C/G SNPs were removed prior to imputation.

All the datasets were phased and imputed using the TopMed Imputation Server (Das et al., 2016). After imputation, variants with a *r* ^*2*^ info score ≤0.75 were excluded. For the statistical analyses, inter‐dataset duplicates (IBD >0.95) were removed from the dataset having the lowest SNP coverage, while, in case of relatedness (IBD >0.0625) the affected or older subjects were kept, independently of SNP coverage.

For association testing analyses, we selected only the Northwestern European subjects since they represented most of the EUR population (approx. 80%) available across the collected GWAS. Analyses were performed using PLINK 2.0 (Chang et al., 2015).

### 2.3 Selection of candidate genomic regions

Genomic regions harboring the genes selected for longevity in previous work (Dato et al., 2018) were queried. Genetic variants from the candidate regions were extracted from the full dataset (approx. 12 million variants), considering their hg19 genomic coordinates (+/− 10 kilobases from the gene boundaries), and further filtered by applying a MAF cut‐off of 0.05 and genotyping rate of 0.95. The final list of variants was generated by Linkage disequilibrium pruning (*r* ^*2*^ > 0.75) to reduce the computational burden of the analysis through the removal of highly correlated variants. Finally, the final dataset comprised a total of 2,469 variants (2,360 SNPs and 109 Ins/dels), as reported in Table S1.

### 2.4 Statistical analyses

For all the analyses, the whole sample was split based on sex and the presence/absence of APOE*4, defining four groups: female APOE*4 carriers, male APOE*4 carriers, female APOE*4 non‐carriers, and male APOE*4 non‐carriers.

After the QC phase, a logistic regression analysis, with an additive model of association, was performed using PLINK 2.0 on the filtered dataset composed of 20,766 subjects (11,038 LOAD cases and 9,728 controls) and 2,469 variants, to estimate single‐marker effects on the predisposition to LOAD. Age, three principal components from the ancestry analysis, APOE*2 dosage, and study site were used as covariates in the regression models. Results from the univariate analysis of the four study groups were meta‐analyzed using GWAMA (Mägi & Morris, 2010). To determine the statistical significance of all the univariate analysis results, a Bonferroni's multiple testing correction was applied [(0.05/2469 variants*4 study groups)], yielding a *p*‐value threshold of 5 × 10^−6^. A nominal *p*‐value < 0.05 was used as a filter for the main effect estimation and for selecting the variants to include in the gene–gene interaction analysis carried out using Multifactor dimensionality reduction (MDR) (Ritchie et al., 2001).

*p*‐value distribution enrichment analysis was performed using Pearson's chi‐square on two‐way contingency tables testing the observed number of variants passing the nominal level of statistical significance (*p* < 0.05) versus the expected one, deeming as statistically significant a Bonferroni's multiple testing correction of *p* < 0.013 (0.05/4 study groups) for individual study group or *p* < 0.05 for the meta‐analysis.

To plot single and common variants between groups of samples, Venn diagrams were created with VennDiagram R Package version 1.7.1 (https://cran.r‐project.org/web/packages/VennDiagram/index.html).

Gene‐based association test was performed by Versatile Gene‐based Association Study‐2 version 2 (VEGAS2, https://vegas2.qimrberghofer.edu.au/) (Mishra & Macgregor, 2015) particularly useful for analyzing GWAS summary statistics. Based on the association *p*‐values of the individual variants, VEGAS2 sums the effects of all the variants within a gene and generates a gene‐based test statistic by doing simulations of the multivariate normal distribution.

The epistatic interaction of up to four bi‐allelic variants was tested using MDR (Ritchie et al., 2001). This methodology estimates high‐order interactions among variants, with respect to a given phenotype also when their individual effect is small to moderate, allowing the discovery of multi‐loci genotype combinations associated with high or low disease risk. An entropy‐based clustering algorithm sets a contingency table for k gene variants and calculates case–control ratios for each of the possible multi‐loci genotypes; a genotype combination is considered high‐level if it is more present in cases compared to controls. For each factor, the MDR interaction model describes percentage of entropy (information gain or IG) and plots a network of two‐way interactions, with positive values of entropy indicating synergistic or non‐additive interaction while negative entropy values indicate redundancy between the markers or lack of any synergistic interaction between them. In the network, red and orange connections indicate non‐linear interactions, green and brown connections indicate independence or additivity, and blue connections indicate redundancy. For all 2‐order, 3‐order, or 4‐order combinations, the best model is considered the one found more consistent in different replicates (expressed as CV, consistency values and accuracy, i.e., training balanced accuracy level). To calculate significance, permutation testing was applied, dividing the data set into 10 portions, and using nine portions as a training data set, and the remaining as a testing data set. Ten thousand permutations were performed, to determine a cutoff threshold for an alpha = 0.05 significance level. For each order of interaction tested, an odds ratio (OR) is outputted, referring to the best combination of variants (best model), while determining the multi‐locus high‐risk combinations.

Multifactor dimensionality reduction analyses were implemented in the open‐source MDR software package version 3.0.2 (https://sourceforge.net/projects/mdr/).

### 2.5 Functional annotation

Functional annotation of risk variants was performed by using multiple bioinformatic tools and databases, including the HaploReg database (https://pubs.broadinstitute.org/mammals/haploreg/haploreg.php), the GTEX portal (https://gtexportal.org/home/), RegulomeDB database (https://beta.regulomedb.org/regulome‐search/) version 2.1, SNP Nexus (https://www.snp‐nexus.org/v4/), and MetaBrain (https://www.metabrain.nl/cis‐eqtls.html). LDlink's LDproxy tool (https://analysistools.nci.nih.gov/LDlink/) (Machiela & Chanock, 2015) and European population data was used for SNP LD analysis.

## 3 RESULTS

### 3.1 Single‐variant and gene‐based analysis

**FIGURE 1:**
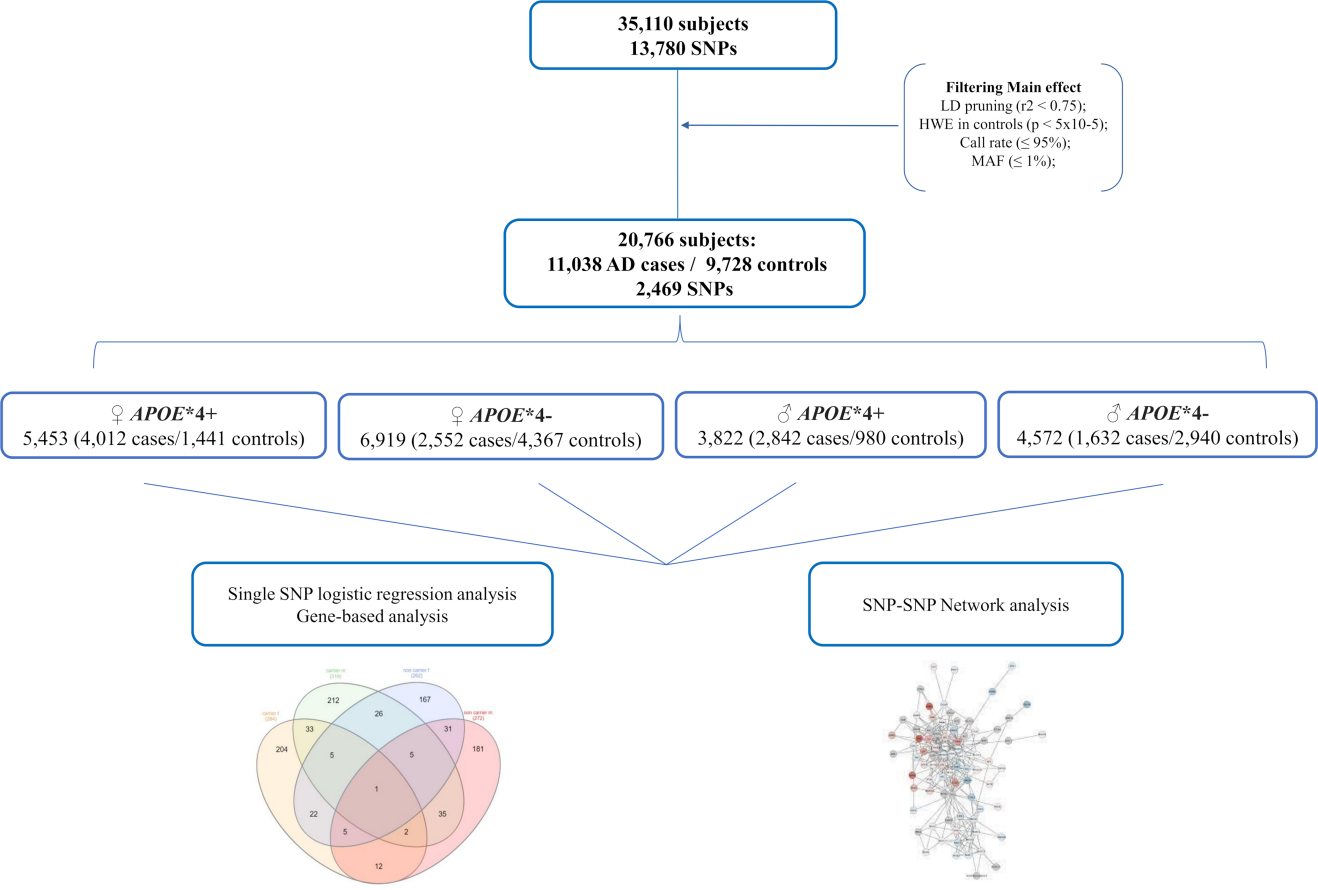
Flowchart describing the steps of the analysis after quality control and filtering.

The flowchart in Figure 1 details the study design. After quality control and filtering, we performed logistic regression analysis on the sample divided in four sub‐groups according to sex‐ and APOE*4 status, and meta‐analyzing them thereafter. In Table S2 we reported the variants associated with the disease risk at a nominal *p*‐value, along with their chromosomal location, the assigned gene and the main functional pathway associated with each coded protein. A graphical representation of all the obtained *p*‐values is presented as a Manhattan plot (Figure S1). These associations, however, did not stand upon correction for multiple comparisons (*p* < 5 x 10^−6^) either when considering the single study group or when meta‐analyzed across the four study groups. Nonetheless, we observed a statistically significant 45% enrichment (*p* = 0.002) in the distribution of nominally significant variants (*p* < 0.05) when the four study groups were meta‐analyzed (Table 2 **)**, even though no statistically significant enrichment was found when analyzing individually the four study groups. Interestingly, we also detected a statistically significant enrichment from the meta‐analysis Cochran's heterogeneity statistic's *p*‐values (*p* = 0.020, Table 2), supporting the existence of APOE*4 and sex‐specific effects.

**TABLE 2:** *p*‐value distribution enrichment analysis performed across the four study groups and the meta‐analysis results.

Group	*N* of variants with *p* < 0.05	OR	Enrichment *p*
APOE*4^+^ females	148 (6.0%)	1.216	0.118
APOE*4^+^ males	157 (6.4%)	1.295	0.036
APOE*4^−^ females	115 (4.7%)	0.932	0.597
APOE*4^−^ males	136 (5.5%)	1.112	0.406
Meta‐analysis			
Meta‐analysis *p*‐value	174 (7.1%)	1.446	**0.002**
Cochran's heterogeneity statistic's *p*‐value	161 (6.5%)	1.331	**0.020**

*Note*: We deemed as statistically significant a Bonferroni's multiple testing correction of *p* < 0.013 (0.05/4 study groups) for individual study group or *p* < 0.05 for the meta‐analysis. *p*‐values reported in bold are statistically significant.

Abbreviations: OR, odds ratio; *p*, *p*‐value.

Within the four study groups, the nominally associated variants belong to 126 different genes, of which 20 are in the IIS, 31 in DNA repair, and 23 in stress response pathways, whereas the remaining 46 are related to other pathways, such as immunity and membrane trafficking (see Table S2). These last genes emerged probably because of a high gene density and/or overlapping genes in some of the genomic regions considered, which extend 10 kb upstream and downstream of the candidate gene boundaries.

**FIGURE 2:**
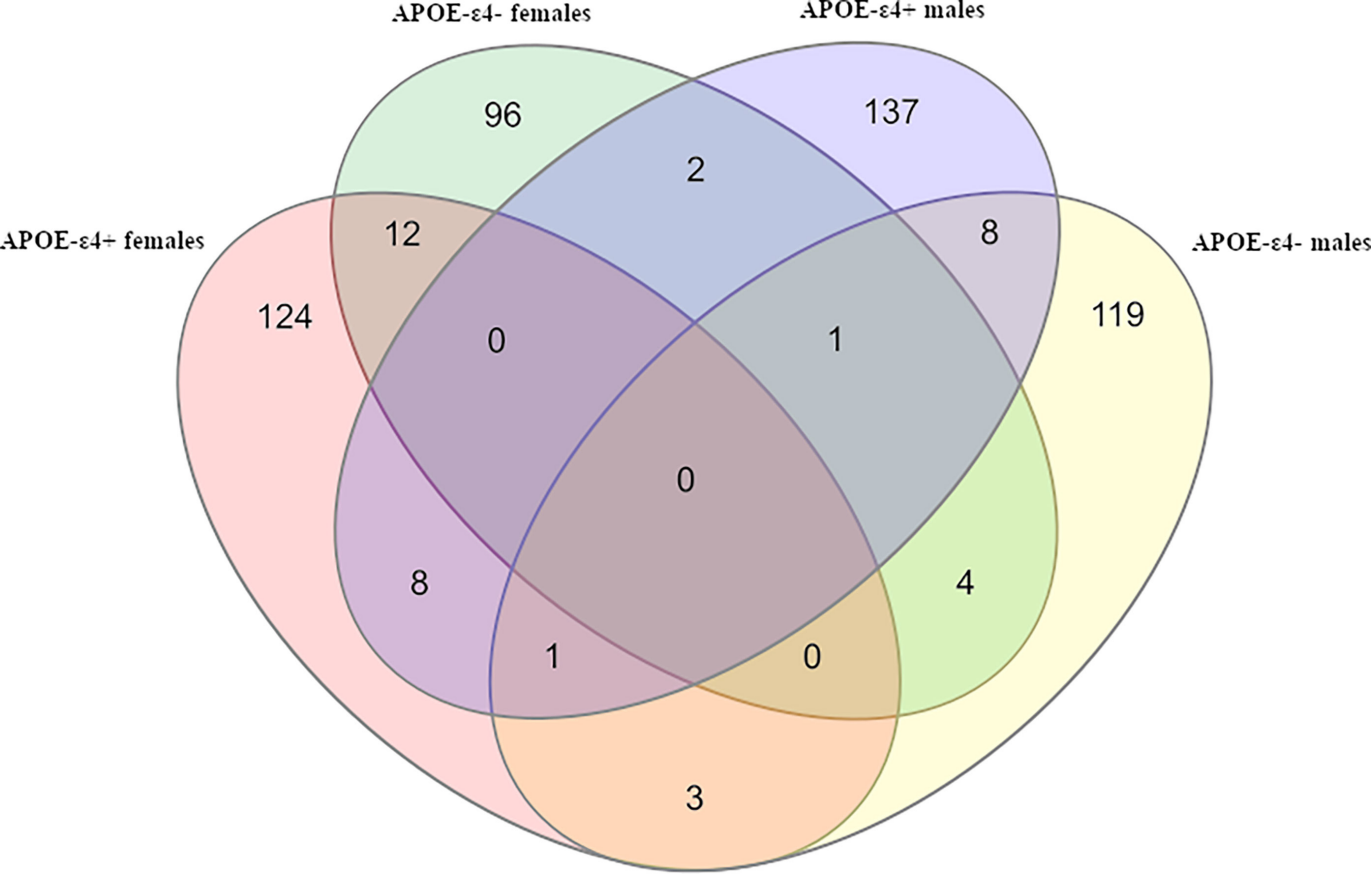
VENN diagram built on Table S1 data, showing the number of variants associated with LOAD in each sub‐group of samples and in common between different groups.

More specifically, the variants significantly associated in the sub‐groups were: 148 in APOE*4^+^ females, 157 in APOE*4^+^ males, 115 in APOE*4^−^ females, and 136 in APOE*4^−^ males. The top‐variants (*p* < 0.001) were: rs17810889‐*C8orf49* and rs5742665‐*IGF1* in APOE*4^+^ females, rs56190996‐*IGF1R* and rs8113762‐*IRGQ* in APOE*4^+^ males, rs28362737‐*AQP1* and rs35519594‐*XDH* in APOE*4^−^ females, rs3729587‐*XPC* and rs142270994‐*CTD‐3094 K11.1* in APOE*4^−^ males. The Venn diagram for the gene variants listed in Table S2 shows the number of variants in each sub‐group and those shared (Figure 2). As it is shown in Table 3, 12 variants were associated with LOAD risk in females, independently from APOE*4 status, five of which showed an opposite direction of effect in the two sub‐groups (Table 3a). In males, eight variants were associated with LOAD independently from APOE*4 status, five of them showing a divergent effect in the two sub‐groups (Table 3b). On the other hand, APOE*4 carriers, independent of sex, share eight markers, five of which show an opposite effect in the two sexes (Table 3c), while APOE*4^−^ share four markers, two of which show an opposite effect in the two sexes (Table 3d).

**TABLE 3:** Gene variants nominally associated with LOAD, shared between: (A) APOE*4^+^ females and APOE*4^−^ females; (B) APOE*4^+^ males and APOE*4^−^ males; (C) APOE*4^+^ females and APOE*4^+^ males; (D) APOE*4^−^ females and APOE*4^−^ males.

Variant	Chr.	Position	Gene SYMBOL	EA	OR	*p*	OR	*p*
**A**					APOE*4 ^***+***^ **females**		APOE*4 ^***−***^ **females**	
rs13183641	5	95,147,287	*RHOBTB3*	T	0.873	0.003	0.909	0.010
rs4561	5	95,152,313	*RHOBTB3*	G	0.886	0.008	0.910	0.011
rs2348974	5	95,143,394	*RHOBTB3*	C	1.119	0.015	1.093	0.014
rs6556881	5	95,134,419	*RHOBTB3*	G	0.908	0.038	0.925	0.034
rs34886287	5	80,077,309	*MSH3*	C	1.110	0.029	1.105	0.007
rs2645429	8	11,660,051	*FDFT1*	A	1.111	0.048	0.902	0.016
rs2645433	8	11,657,921	*RP11‐297 N6.4*	C	0.902	0.027	1.081	0.035
rs112267867	9	118,905,353	*PAPPA*	A	1.260	0.037	1.179	0.044
rs6583817	10	94,247,247	*IDE*	T	0.836	0.006	0.859	0.005
rs4752254	10	120,910,136	*SFXN4*	C	0.895	0.042	1.094	0.036
rs2860173	19	7,129,086	*INSR*	A	1.230	0.016	0.870	0.039
rs12979722	19	7,118,878	*INSR*	C	1.169	0.042	0.846	0.006
**B**					APOE*4 ^***+***^ **males**		APOE*4 ^***−***^ **males**	
rs78686161	1	242,065,356	*EXO1*	A	1.450	0.005	1.303	0.006
rs670548	6	53,366,989	*GCLC*	C	0.859	0.009	1.123	0.015
rs7740677	6	46,649,231	*SLC25A27*	C	1.167	0.030	1.154	0.013
rs17069665	6	108,941,468	*FOXO3*	G	0.832	0.033	1.169	0.031
rs2684794	15	99,484,953	*IGF1R*	C	0.848	0.038	1.135	0.049
rs7245548	19	45,981,840	*ERCC1*	T	1.126	0.036	0.912	0.049
rs4803825	19	45,986,483	*RTN2*	G	0.889	0.042	1.128	0.012
rs4328554	19	7,249,830	*INSR*	T	1.129	0.046	1.130	0.014
**C**					APOE*4 ^***+***^ **females**		APOE*4 ^***+***^ **males**	
rs17882672	6	53,408,883	*GCLC*	T	0.858	0.018	1.236	0.018
rs7092649	10	60,005,202	*IPMK*	A	0.878	0.017	0.866	0.037
rs17636964	10	59,953,046	*IPMK*	C	1.139	0.025	1.167	0.029
rs1625716	10	59,960,083	*IPMK*	G	0.826	0.038	0.784	0.033
rs7138318	12	104,736,394	*TXNRD1*	C	0.861	0.002	1.146	0.024
rs7979495	12	104,625,779	*TXNRD1*	G	0.810	0.018	1.247	0.047
rs141516621	19	7,280,152	*INSR*	A	1.232	0.003	0.844	0.043
rs7258382	19	7,262,569	*INSR*	C	1.141	0.048	0.841	0.027
**D**					APOE*4 ^***−***^ **females**		APOE*4 ^***−***^ **males**	
rs2010704	2	31,622,465	*XDH*	A	1.098	0.011	1.106	0.030
rs147249797	2	31,601,541	*XDH*	T	1.092	0.034	1.123	0.029
s10759223	9	110,065,158	*RAD23B*	C	1.078	0.038	0.909	0.041
rs2494741	14	105,249,322	*AKT1*	T	1.157	0.032	0.754	0.002

Abbreviations: EA, effect allele; OR, Odds Ratio; *p*, *p*‐value.

**FIGURE 3:**
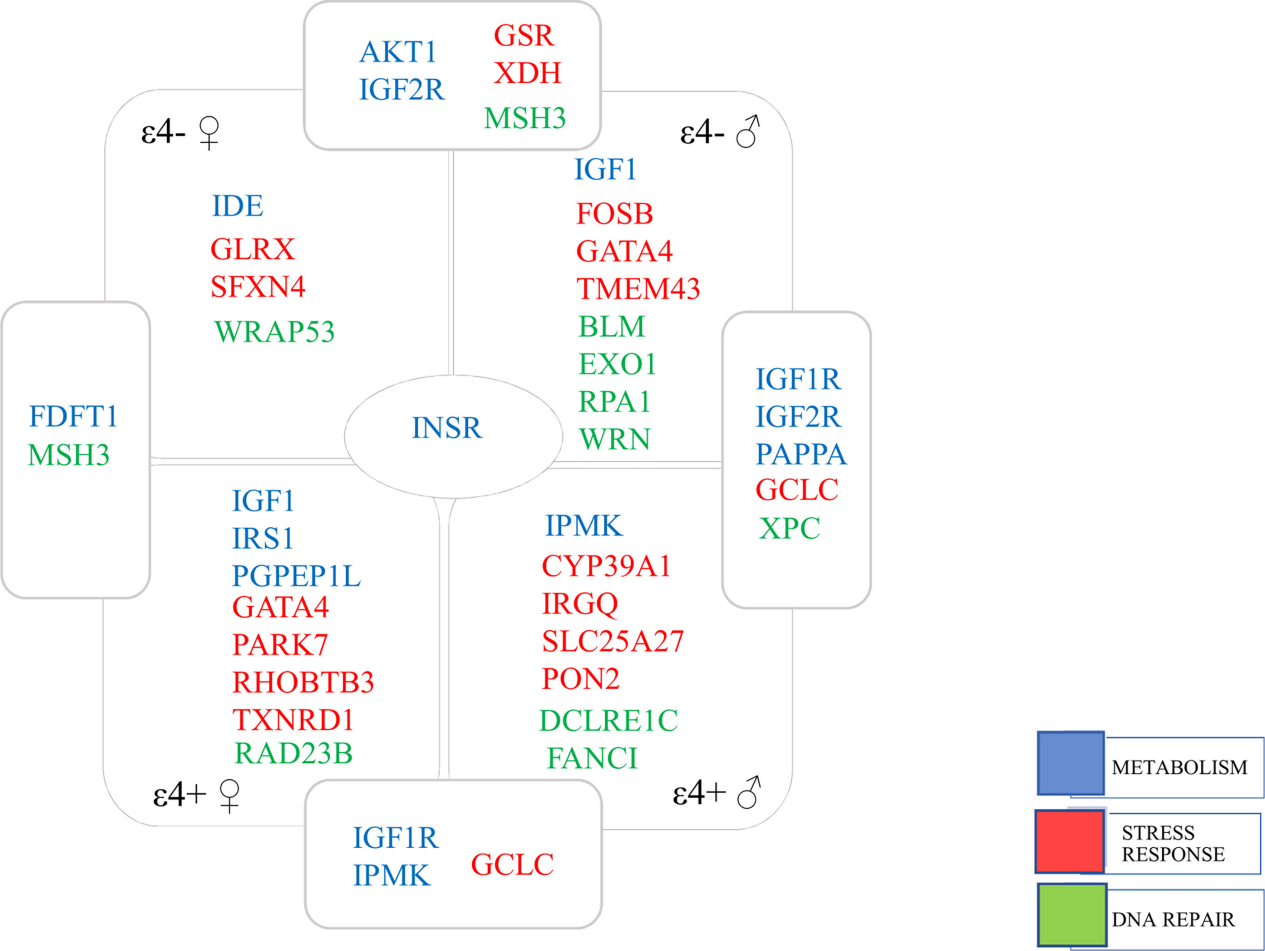
Schematic representation of gene‐based analysis, reporting the top‐genes (*p* < 0.01) associated with the disease in each different sub‐group and those shared. Colors represent the three analyzed pathways.

For testing the enrichment of associated variants in the same gene, we then performed a gene‐based analysis using VEGAS2. Table S3 reports the top‐genes (*p* < 0.01) and the top‐variant associated with the disease risk in the four sub‐groups of samples. The schematic representation of Table S3 data is reported in Figure 3. The *INSR* gene is the only one common to all the sub‐groups, although through different variants. *FDFT1* and *MSH3* were the top‐genes in females, while *IGF1R*, *IGF2R*, *PAPPA*, *GCLC*, and *XPC* genes were the top‐genes in males, independent of APOE*4 status. These genes, thus, appear to be associated with LOAD in a sex‐specific way. Conversely, APOE*4+ subjects, independent of by sex, share markers in *IGF1R*, *GCLC*, and *IPMK* genes; APOE*4^−^ subjects, by contrast show an enrichment of significant markers in *IGF2R*, *AKT1*, *GSR*, *MSH3*, and *XDH* genes, suggesting them as influenced by *APOE* status in LOAD susceptibility.

### 3.2 Analysis of epistatic interactions

**FIGURE 4:**
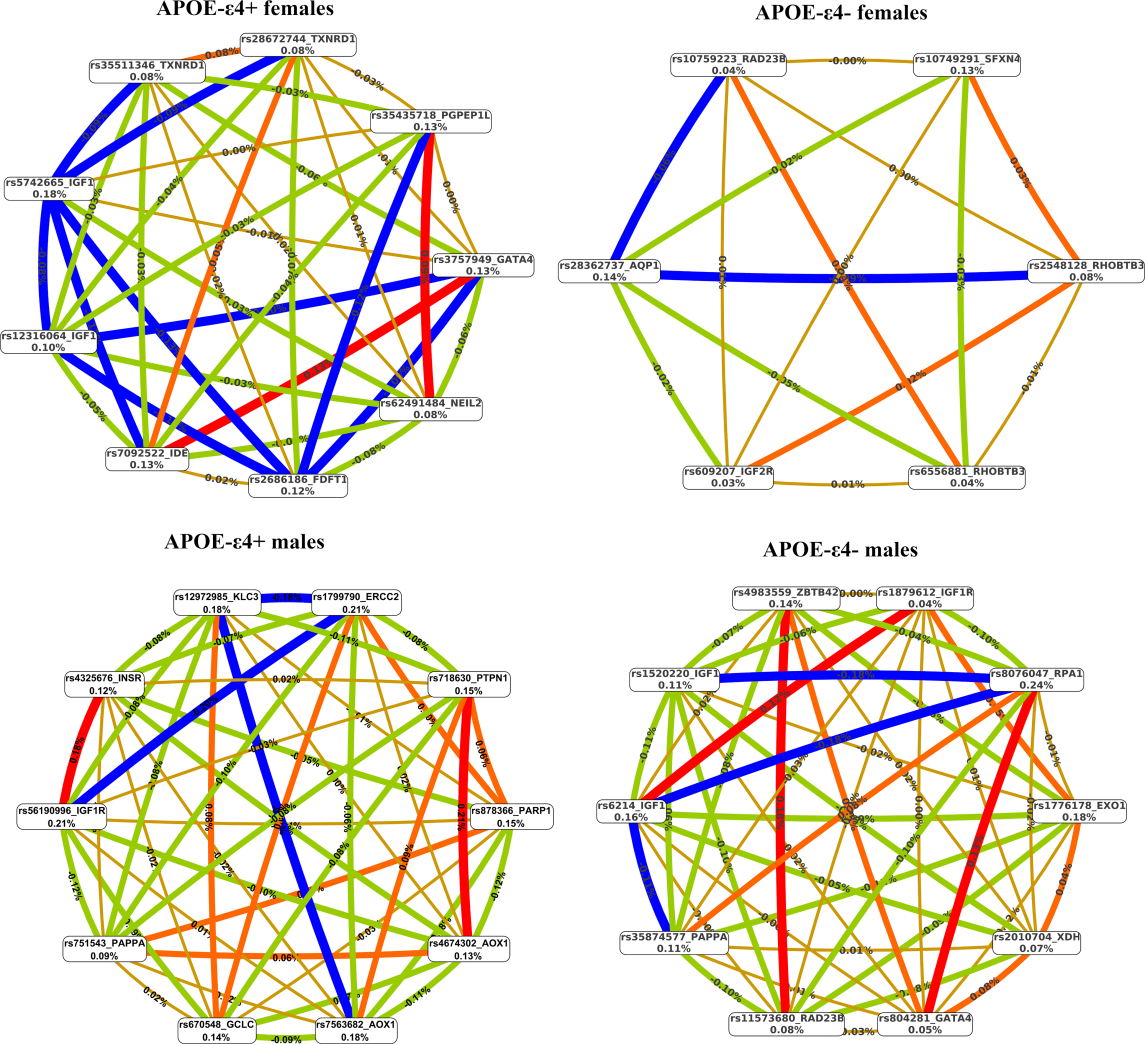
Interaction graphs, reporting the significant markers from MDR analysis, in the group of APOE*4^+^ females (a), in APOE*4^−^ females, in APOE*4^+^ males, and in APOE*4^−^ males (d). For each variant we reported the value of information gain (IG) in per cent, while numbers in the connections indicate the entropy‐based IG for the variant pairs. Red bar and orange bar indicate the high‐level synergies on the phenotype, while the brown indicate a medium‐level interaction, green and blue connections with negative IG values indicate redundancy or lack of synergistic interactions between the markers.

With the aim of finding gene–gene epistatic interactions between 2‐ or among 3‐ and 4‐markers associated with the disease risk, we used the MDR approach. Table 4 shows the gene–gene interactions for LOAD resulting from the analysis, while Figure 4 depicts the interaction networks between variants in each sub‐group.

**TABLE 4:** Significant results of gene–gene interaction associations, resulting from the MDR analysis.

	Gene–gene combination	Genes included in the combination	Pathways involved	OR(95% CI.)	*p*‐value (10,000 permutations)			
					APOE*4^+^ females	APOE*4^+^ males	APOE*4^−^ females	APOE*4^−^ males
A: APOE*4 ^**+**^ **females**								
2‐order interactions	rs3757949/rs7092522	*GATA4*/*IDE*	Stress response/Metabolism	1.42 (1.25, 1.62)	**0.0001**	0.1945	0.7051	0.6106
	rs62491484/rs35435718	*NEIL2*/*PGPEP1L*	DNA repair/Stress response	1.36 (1.19, 1.54)	**0.0002**	0.6143	0.8744	**0.0294**
3‐ and 4‐order interactions	rs62491484/rs28672744/rs35435718	*NEIL2*/*TXNRD1*/*PGPEP1L*	DNA repair/Stress response	1.56 (1.38, 1.76)	**0.0001**	0.8034	0.9658	0.4729
	rs2686186/rs7092522/rs12316064/rs35511346	*FDFT1*/*IDE*/*IGF1*/*TXNRD1*	Stress response/Metabolism	1.96 (1.72, 2.22)	**0.0001**	0.9389	0.6944	0.8240
B: APOE*4 ^**+**^ **males**								
2‐order interactions	rs4674302/rs718630	*AOX1*/*PTPN1*	Stress response/Metabolism	1.46 (1.27, 1.69)	0.8127	**0.0001**	0.9709	0.9489
	rs56190996/rs4325676	*IGF1R*/*INSR*	Metabolism	1.49 (1.28, 1.73)	0.5017	**0.0002**	0.2057	0.2499
3‐ order interaction	rs7563682/rs670548/rs12972985	*AOX1*/*GCLC*/*KLC3*	Stress response/Metabolism	1.69 (1.46,1.96)	0.9976	**0.0001**	0.9992	0.2060
C: APOE*4 ^**−**^ **males**								
2‐order interactions	rs11573680/rs4983559	*RAD23B*/*ZBTB42*	DNA repair	1.43 (1.24, 1.64)	0.7629	**0.0318**	0.7856	**0.0004**
	rs6214/rs1879612	*IGF1*/*IGF1R*	Metabolism	1.33 (1.18, 1.50)	0.6723	0.8911	0.5558	**0.0004**
3‐ and 4‐order interactions	rs11573680/rs1520220/rs4983559	*RAD23B*/*IGF1*/*ZBTB42*	DNA repair/Metabolism	1.55 (1.37, 1.76)	0.2953	0.8032	0.9899	**0.0001**
	rs1776178/rs2010704/rs804281/rs1879612	*EXO1*/*XDH*/*GATA4*/*IGF1R*	DNA repair/Stress response/Metabolism	1.16 (1.09, 1.40)	0.5058	0.9903	0.9301	**0.0003**

*Note*: 2‐ 3‐ and 4‐loci interactions are shown, with respective pathways, selected for CV (10/10 replicates) and training balanced accuracy (>0.5). The OR value of the best combination of variants (best model) is reported. *p*‐values reported in bold are considered statistically significant.

In APOE*4^+^ females, two 2‐order epistatic interactions (Figure 4a) were found significantly associated with LOAD, namely the combinations rs3757949‐*GATA4*/rs7092522‐*IDE* and rs62491484‐*NEIL2*/rs35435718‐*PGPEP1L*. These four variants were among the most significant associated with LOAD (*p* < 0.01) in this sub‐group for the single‐variant analysis. Both epistatic interactions were inter‐pathway, combining markers of genes belonging, respectively, to stress response (*GATA4*) and metabolism (*IDE*), and to DNA repair (*NEIL2*) and stress response (*PGPEP1L*). The same pathways also emerged from the 3‐ and 4‐order loci interactions (Table 4a), where two markers of the gene *TXNRD1* (rs28672744 in the 3‐order and rs35511346 in the 4‐order interactions) and one of *FDFT1* (rs26861869) are included. As indicated by the interaction graph in Figure 4a, several variants show a redundant effect (blue lines) in the network. Among them, the rs5742665‐*IGF1* is the top‐variant from the gene‐based analysis in this sub‐group (*p* < 0.001; Table S2) and seems to be a LOAD specific marker of APOE*4^+^ females, not being associated with disease in the other sub‐groups.

No significant epistatic interactions were observed in the group of APOE*4^−^ females (Figure 4b). The variant rs28362737‐*AQP1* present in the network is the most associated with LOAD in this group, but it seems to have a univariate effect.

In APOE*4^+^ males (Figure 4c), the most important gene–gene interactions (red lines) were: rs4674302‐*AOX1*/rs718630‐*PTPN1* and rs56190996‐*IGF1R*/rs4325676‐*INSR*. These interactions are driven by rs718630‐*PTPN1* and rs56190996‐*IGF1R* variants, which are the most significantly associated with LOAD (*p* < 0.01) in the single‐marker analysis, with the last representing the top‐variant in *IGF1R* associated with LOAD in the subgroup. Both interactions are intra‐pathway epistasis between genes involved in metabolism. The 3‐order interaction is again an intra‐pathway epistasis, involving *AOX1*, *GCLC*, and *KCL3* genes whose products act in metabolism.

In APOE*4− males (Figure 4d), two gene–gene interactions resulted from MDR analysis namely rs11573680‐*RAD32B*/rs4983559‐*ZBTB42*, relative to the DNA‐repair pathway, and rs6214‐*IGF1*/rs1879612‐*IGF1R* to metabolism. 3‐order and 4‐order interactions resume the collaboration of DNA repair and metabolism, in addition to stress response pathway, in the susceptibility to LOAD in this sub‐group.

To determine whether the gene–gene interactions found were exclusive between the four sub‐groups, we ran MDR using those selected interactions across all the groups. Notably, we observed that the rs62491484‐*NEIL2*/rs35435718‐*PGPEP1L* epistatic interaction found in APOE*4^+^ females occurred also in APOE*4^−^ males (*p* = 0.028) (Table 4). However, the high‐risk genotypic combinations were not comparable (Figure S2). Similarly, the rs11573680‐*RAD32B*/rs4983559‐*ZBTB42* interaction found in APOE*4^−^ males occurred also in the APOE*4^+^ males (*p* = 0.038) (Table 4), although with a different pattern of high‐risk genotypic combinations (Figure S2).

### 3.3 Functional annotation analysis

Next, we performed functional annotation to ascertain biological significance of the variants identified in the single variant and interaction analyses. To this end, several databases and tools were considered, as reported in the Materials & Methods section. Table S4 reports the most relevant results of eQTL analysis of the associated variants and their proxies in high LD (*r* ^*2*^ > =0.8) According to this analysis some of the risk variants act as cis‐eQTL regulatory elements which modulate the expression of the corresponding gene or of nearby genes in specific brain regions. Moreover, evidence of a regulatory role (1f or 1b score in RegulomeDb and association with regulatory elements in SNPnexus) was found for other variants associated with the disease phenotype.

Some variants reported a relevant number of proxies in LD (*r* ^*2*^ ≥ 0.8), although none showed evidence of stronger regulatory potential than the lead variant. As reported in Table S4, the significant variants we identified were not previously implicated in LOAD, yet some of them have been reported to affect some age‐related disorders.

## 4 DISCUSSION

The genetic architecture of LOAD has been widely studied in recent years, and so far, 100 of risk genes and related rare and common genetic variants have been identified, but many remain to be uncovered (Andrews et al., 2023). As advanced age is the greatest risk factor for LOAD, shared genetic pathways between LOAD and longevity are expected, although their connections are still not fully understood (Tesi et al., 2021). Phenotypic heterogeneity (i.e., distinct groups of subjects present with different clinical syndromes) and/or temporal heterogeneity (i.e., substantial inter‐subject variance in age‐at‐onset and rate of decline) (Young et al., 2018) are common in LOAD. Another challenge is the small contribution of individual genetic variants in complex phenotypes. Moreover, several studies have emphasized that genetic interactions may be more important than single markers in neurodegenerative diseases and longevity (Gilbert‐Diamond & Moore, 2011), suggesting the existence of non‐additive heritability to these traits.

In this study, we applied a gene–gene interaction approach to a set of genetic variants selected for being on or near genes involved in the IIS, the DNA repair, and the oxidative stress response pathways, previously linked to human longevity (Dato et al., 2018). To identify gene signatures for LOAD related to sex and/or to *APOE* genotype, we performed analyses stratified by APOE*4 status and sex. The validity of the choice of such study design was underscored by additional cross‐validation analyses, which confirmed the presence of heterogeneity across the different sub‐groups.

Overall, this study highlights two aspects of the genetic complexity in LOAD. First, it supports the claim of shared genetic pathways between longevity and LOAD. Second, it suggests that sex and *APOE* genotype can drive different genetic risk factors. This evidence appeared clear in single variant analyses. In APOE*4^+^ females, the two top variants were rs17810889 upstream of *C8orf49*, and rs5742665 in an intron of *IGF1* gene, one of the master genes in IIS metabolic pathway. In APOE*4^−^ females, the top‐variant was rs28362737, an intronic variant of *AQP1*, the gene encoding Aquaporin 1, a protein associated with amyloid‐beta deposition in AD brains (Misawa et al., 2008). In APOE*4^+^males, the top‐variants were rs56190996, in an intron of *IGF1R* (insulin‐like growth factor receptor), and rs8113762 located at the 3’‐UTR of *IRGQ* (immunity‐related GTPase Q), while in APOE*4^−^ subjects, the most significant variant was rs3729587 in the DNA repair gene *XPC* (Xeroderma pigmentosum, complementation group C). Many of these markers appear to be functionally relevant, being cis‐eQTL or trans‐eQTL, and specifically in brain regions, as reported in Table S4. However, we cannot exclude that one of their proxies in LD (*r* ^*2*^ ≥ 0.8) could be the actual causal variant, although none showed a potential regulatory effect higher than the associated variants.

It is also worth noting that some of the associated variants showed an opposite effect in the two sexes. This is line with several studies demonstrating that some variants, the so‐called “sexually antagonistic variants”, have a beneficial effect in one sex but deleterious (or null) effects in the other, thus having a role in shaping differences between males and females in age‐related disease outcomes as well as in survival (Iannuzzi et al., 2023; Lagou et al., 2021).

Results from gene‐based analysis further highlighted genes associated with LOAD in a sex‐ and *APOE*‐specific manner. *INSR*, is the only one gene shared among the four sub‐groups of patients, which encodes the insulin receptor, an important component of the insulin pathway. Through its binding with insulin, INSR controls the glucose metabolism in the brain helping to maintain neuronal functioning (de la Monte & Wands, 2005). Decline in glucose metabolism is indeed one of the earliest and most common anomalies observed in patients with LOAD (Akhtar & Sah, 2020). A recent study by Leclerc et al. (2023) reported that, in association with β‐amyloid pathology, defects in the activation of *INSR* at the blood–brain barrier strongly contribute to brain insulin resistance in LOAD. Genetic variants of this gene were found enriched in centenarians, thus indicating *INSR* a key mediator of human longevity (Barbieri et al., 2003).

The evaluation of the joint effect of different markers through gene–gene interactions further added insights into the genetic architecture of LOAD. Among the significantly interacting genes, *PTPN1* (protein tyrosine phosphatase non‐receptor type 1, IIS pathway), *TXNRD1* (thioredoxin reductase 1; stress response), and *IGF1R* (insulin‐like growth factor 1 receptor; IIS) were already found in combinations affecting survival to old age (Dato et al., 2018; Ukraintseva et al., 2021). *PTPN1* and *TXNRD1* were engaged in best risk combinations for longevity, respectively, with *IGF1R* and *TP53* (DNA repair), while *IGF1R*, interacted with *TP53* and *TGFBR2* (cell proliferation). In LOAD *PTPN1* interacts with a partner from the stress response pathway, like *AOX1*. Notably, *TXNRD1* is engaged in 4‐order risk combination for LOAD with genes from the IIS pathway, like *IGF1*, *FDFT1* (farnesyl‐diphosphate farnesyltransferase 1), and *IDE* (insulin degrading enzyme). Similarly, *IGF1R* is associated with the disease in a 4‐order synergic combination with *EXO1* (DNA repair), *XDH* (stress response), and *GATA4* (IIS), behind its ligand *IGF1*. Overall, these findings suggest that in LOAD as in longevity *TXNRD1* and even more *IGF1R*, may represent hubs interconnecting multiple signaling pathways. The different sub‐processes may instead explain the sex‐ and *APOE*‐specific associations we found.

Taken together, these results suggest that longevity loci may also drive LOAD neuropathology through *APOE*‐ and sex‐related specific gene–gene interactions. The complex interplay among sex, *APOE*, and age may influence the severity and the temporal trajectory of LOAD progression, creating a risk profile for LOAD that could serve to identify high‐risk individuals (Riedel et al., 2016). Mechanisms underlying the sex differences are unknown; the literature largely supports the claim that it may be due to the known differences in longevity between men and women (Hossin, 2021), while some propose that sex dimorphisms in stress responses can contribute to the increased prevalence of LOAD in women (Yan et al., 2018). Sex divergence in biochemical responses to stress were reported along the hypothalamic–pituitary–adrenal axis and in the activation of the cortical corticotrophin‐releasing factor receptor 1 signaling pathway, leading to distinct female‐biased increases in molecules associated with LOAD pathogenesis (Yan et al., 2018). In our analysis, effectors of stress response, such as *TXNRD1* and *GATA4*, have been found in all the sub‐groups of patients, suggesting that an impaired ability to induce a stress response represents an underlying risk factor for LOAD. Interestingly, in APOE*4^+^ females, potentially experiencing higher levels of hydroxyl radicals and reduced levels of mitochondrial antioxidants compared to non‐APOE*4 carriers (Ihara et al., 2000), all significant epistatic interactions were among genes involved in oxidative stress and those belonging to the other pathways.

## 5 CONCLUSIONS

Collectively, the present results indicate that several loci repeatedly implicated in aging and longevity also contribute to late‐onset Alzheimer's disease (LOAD) risk. Most of the top genes associated are assigned to pathways related to metabolism, highlighting their relevance both in the aging process and the pathological events leading to LOAD. Interestingly, this study bolsters the evidence of specific interactions among established risk factors for LOAD, that is *APOE* genotype and sex, and these genes. This suggests that different trajectories of cognitive aging may be the result of specific epistatic effects between genetic and non‐genetic risk factors. Although our conclusions are based on the evidence of “statistical epistasis”, which magnitude and contribution to the variance of complex traits is a highly debated topic (Hivert et al., 2021), in accordance with other authors (Singhal et al., 2023) we think that the potential for “functional epistasis” to drive expressivity and explain clinical heterogeneity in complex diseases is mounting. In the complex scenario of LOAD, understanding the functional changes associated with different combinations of interacting entities (SNPs, genes, pathways, etc.) may help to disentangle the genetic architecture underlying disease development, and interindividual differences that underpin disease, finally leading to precision medicine approaches for early detection of individuals at higher risk for cognitive decline or dementia.

## AUTHOR CONTRIBUTIONS

Idea/concept: SD, FDR, GR, VN; Data curation/processing: VN, SP, MEB, YLG; Data analysis: SD, FDR, PC, GR, VN; Writing: SD, FDR, PC, GR, VN; Advice: MEB, YLG, MDG, GP.

## CONFLICT OF INTEREST STATEMENT

The authors declare no competing interests.

## Supporting information


Figure S1.

Figure S2.

Table S1.

Table S2.

Table S3.

Table S4.

## Data Availability

Data sharing is not applicable to this article as no new data were created or analyzed in this study.
