# Supplementary figures and images for: Sex‐ and APOE‐specific genetic risk factors for late‐onset Alzheimer's disease: Evidence from gene–gene interaction of longevity‐related loci
Source: Aging Cell. 2023 Aug 24;22(9):e13938. doi: 10.1111/acel.13938 (PMC10497850; doi:10.1111/acel.13938)

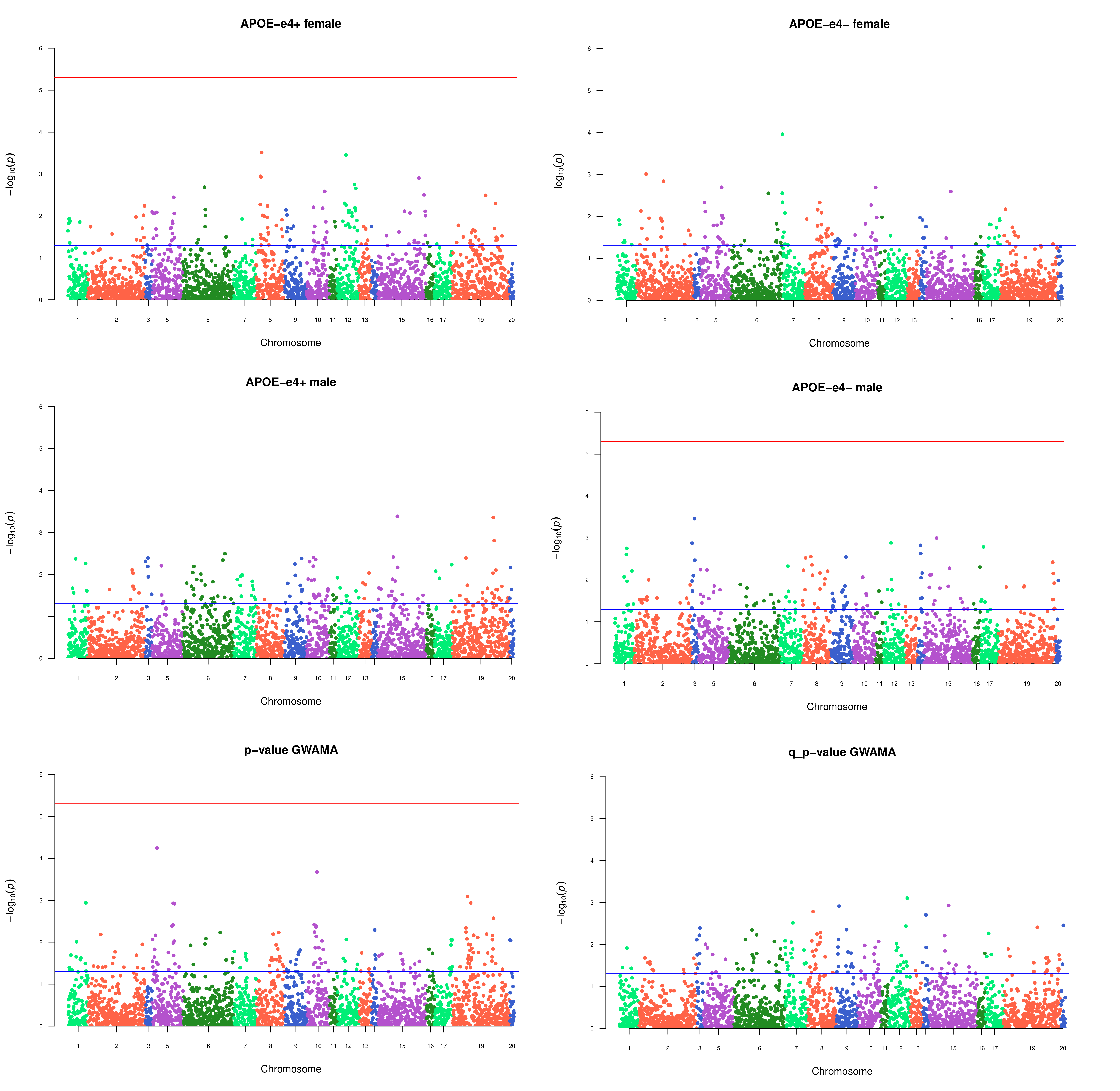

Supplement: Supplementary file 1 — Figure S1. [file ACEL-22-e13938-s004.tiff]

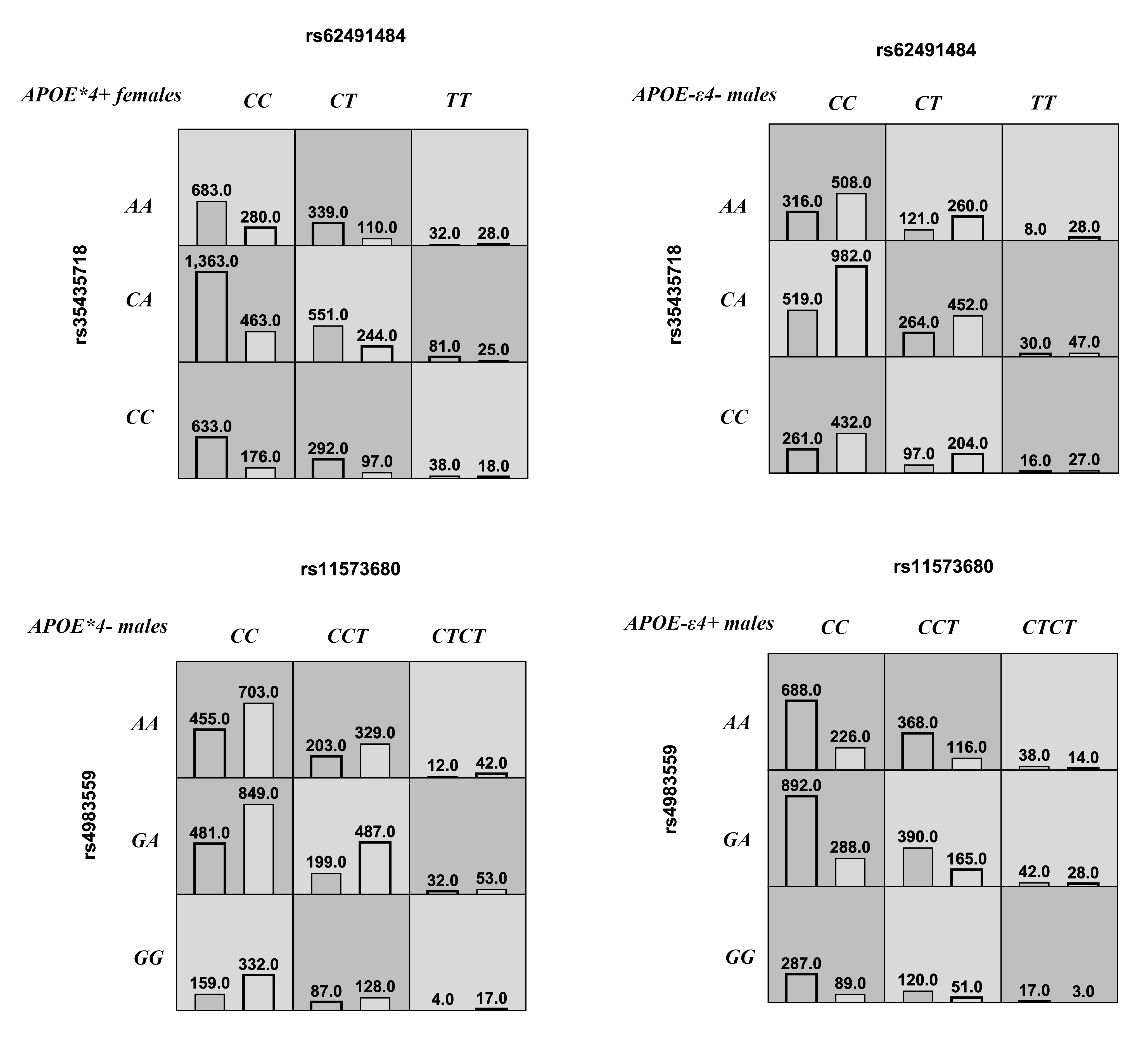

Supplement: Supplementary file 2 — Figure S2. [file ACEL-22-e13938-s003.tiff]
