# Supplementary material for: Sex‐ and APOE‐specific genetic risk factors for late‐onset Alzheimer's disease: Evidence from gene–gene interaction of longevity‐related loci
Source: Aging Cell. 2023 Aug 24;22(9):e13938. doi: 10.1111/acel.13938 (PMC10497850; doi:10.1111/acel.13938)
Supplement: Supplementary file 5 — Table S3. [file ACEL-22-e13938-s001.docx]

**Table S3. Results of gene-based analysis (P < 0.01) and the corresponding top-variant associated with LOAD in the four different sample groups.**

| 1. ***APOE**4^+^ females** |  |
| --- | --- |

| **Chr** | **Gene** | **Variants (N)** | **Start** | **Stop** | **P** | **Top Variant** | **Top Variant P** |
| --- | --- | --- | --- | --- | --- | --- | --- |
| 15 | *IGF1R* | 8 | 99,191,767 | 99,507,759 | 9.099*10^-5^ | rs2684792 | 0.0013 |
| 12 | *TXNRD1* | 8 | 104,609,556 | 104,744,085 | 0.0002 | rs10861185 | 0.0018 |
| 19 | *INSR* | 13 | 7,112,265 | 7,294,011 | 0.0003 | rs141516621 | 0.0032 |
| 8 | *GATA4* | 4 | 11,561,716 | 11,617,509 | 0.0004 | rs3757949 | 0.0011 |
| 6 | *GCLC* | 2 | 53,362,139 | 53,409,927 | 0.0006 | rs72942529 | 0.0021 |
| 15 | *PGPEP1L* | 4 | 99,511,458 | 99,551,024 | 0.0007 | rs56247483 | 0.0031 |
| 12 | *IGF1* | 3 | 102,789,644 | 102,874,378 | 0.0007 | rs5742665 | 0.0004 |
| 5 | *MSH3* | 5 | 79,950,466 | 80,172,634 | 0.0011 | rs4703818 | 0.0082 |
| 2 | *IRS1* | 2 | 227,596,032 | 227,663,506 | 0.0022 | rs2234931 | 0.0096 |
| 8 | *FDFT1* | 7 | 11,653,081 | 11,696,818 | 0.0025 | rs2645438 | 0.0058 |
| 5 | *RHOBTB3* | 3 | 95,066,849 | 95,132,071 | 0.0052 | rs6815 | 0.0191 |
| 10 | *IPMK* | 4 | 59,951,277 | 60,027,694 | 0.0060 | rs7092649 | 0.0176 |
| 1 | *PARK7* | 3 | 8,021,713 | 8,045,342 | 0.0070 | rs226252 | 0.0116 |
| 9 | *RAD23B* | 2 | 110,045,516 | 110,094,475 | 0.0088 | rs7026973 | 0.0094 |

1. ***APOE**4^-^ females**

| **Chr** | **Gene** | **Variants (N)** | **Start** | **Stop** | **P** | **Top Variant** | **Top Variant P** |
| --- | --- | --- | --- | --- | --- | --- | --- |
| 2 | *XDH* | 2 | 31,557,187 | 31,637,611 | 0.0003 | rs35519594 | 0.0010 |
| 19 | *INSR* | 9 | 7,112,265 | 7,294,011 | 0.0010 | rs12979722 | 0.0067 |
| 5 | *MSH3* | 3 | 79,950,466 | 80,172,634 | 0.0023 | rs6151792 | 0.0047 |
| 10 | *SFXN4* | 3 | 120,900,424 | 120,925,204 | 0.0029 | rs10749291 | 0.0021 |
| 8 | *GSR* | 5 | 30,535,579 | 30,585,486 | 0.0052 | rs3779647 | 0.0082 |
| 5 | *GLRX* | 3 | 95,149,552 | 95,158,577 | 0.0052 | rs4561 | 0.0110 |
| 8 | *FDFT1* | 3 | 11,653,081 | 11,696,818 | 0.0065 | rs2252567 | 0.0070 |
| 6 | *IGF2R* | 5 | 160,390,130 | 160,527,583 | 0.0072 | rs6909681 | 0.0151 |
| 14 | *AKT1* | 2 | 105,235,686 | 105,262,080 | 0.0090 | rs2494739 | 0.0123 |
| 10 | *IDE* | 3 | 94,211,440 | 94,333,852 | 0.0092 | rs6583817 | 0.0054 |
| 17 | *WRAP53* | 3 | 7,589,388 | 7,606,820 | 0.0099 | rs2287499 | 0.0116 |

1. ***APOE**4^+^ males**

| **Chr** | **Gene** | **Variants (N)** | **Start** | **Stop** | **P** | **Top Variant** | **Top Variant P** |
| --- | --- | --- | --- | --- | --- | --- | --- |
| 15 | *IGF1R* | 8 | 99,191,767 | 99,507,759 | 0.0001 | rs56190996 | 0.0004 |
| 9 | *PAPPA* | 6 | 118,916,070 | 119,164,600 | 0.0002 | rs7847346 | 0.0057 |
| 19 | *INSR* | 8 | 7,112,265 | 7,294,011 | 0.0003 | rs3815901 | 0.0041 |
| 19 | *IRGQ* | 2 | 44,088,518 | 44,100,287 | 0.0006 | rs8113762 | 0.0004 |
| 6 | *CYP39A1* | 3 | 46,517,316 | 46,620,567 | 0.0015 | rs12215229 | 0.0091 |
| 3 | *XPC* | 3 | 14,186,647 | 14,220,172 | 0.0016 | rs3731125 | 0.0041 |
| 6 | *IGF2R* | 4 | 160,390,130 | 160,527,583 | 0.0020 | rs35646088 | 0.0032 |
| 6 | *GCLC* | 3 | 53,362,139 | 53,409,927 | 0.0023 | rs670548 | 0.0099 |
| 10 | *DCLRE1C* | 4 | 14,946,609 | 14,996,431 | 0.0030 | rs12245497 | 0.0049 |
| 7 | *PON2* | 3 | 95,034,173 | 95,064,384 | 0.0030 | rs7785039 | 0.0146 |
| 10 | *IPMK* | 5 | 59,951,277 | 60,027,694 | 0.0033 | rs7899961 | 0.0044 |
| 6 | *SLC25A27* | 2 | 46,620,651 | 46,645,927 | 0.0034 | rs2270450 | 0.0064 |
| 15 | *FANCI* | 2 | 89,787,193 | 89,860,362 | 0.0083 | rs3087374 | 0.0210 |

1. ***APOE**4^-^ males**

| Chr | Gene | **Variants (N)** | **Start** | **Stop** | **P** | **Top Variant** | **Top Variant P** |
| --- | --- | --- | --- | --- | --- | --- | --- |
| 15 | *BLM* | 3 | 91,260,557 | 91,358,692 | 0.0003 | rs117988672 | 0.0075 |
| 15 | *IGF1R* | 7 | 99,191,767 | 99,507,759 | 0.0003 | rs62023609 | 0.0053 |
| 14 | *AKT1* | 5 | 105,235,686 | 105,262,080 | 0.0003 | rs2498791 | 0.0015 |
| 3 | *XPC* | 3 | 14,186,647 | 14,220,172 | 0.0004 | rs3729587 | 0.0003 |
| 19 | *FOSB* | 2 | 45,971,252 | 45,978,437 | 0.0005 | rs11667571 | 0.0038 |
| 9 | *PAPPA* | 8 | 118,916,070 | 119,164,600 | 0.0006 | rs35874577 | 0.0029 |
| 3 | *TMEM43* | 4 | 14,166,439 | 14,185,180 | 0.0010 | rs4685075 | 0.0013 |
| 12 | *IGF1* | 4 | 102,789,644 | 102,874,378 | 0.0010 | rs6214 | 0.0013 |
| 8 | *WRN* | 2 | 30,890,777 | 31,031,277 | 0.0013 | rs6982140 | 0.0093 |
| 8 | *GATA4* | 5 | 11,561,716 | 11,617,509 | 0.0020 | rs3729851 | 0.0030 |
| 17 | *RPA1* | 2 | 1,733,272 | 1,802,848 | 0.0021 | rs8076047 | 0.0016 |
| 19 | *INSR* | 2 | 7,112,265 | 7,294,011 | 0.0026 | rs2081881 | 0.0146 |
| 2 | *XDH* | 5 | 31,557,187 | 31,637,611 | 0.0034 | rs1346644 | 0.0255 |
| 6 | *IGF2R* | 3 | 160,390,130 | 160,527,583 | 0.0044 | rs77153348 | 0.0223 |
| 5 | *MSH3* | 3 | 79,950,466 | 80,172,634 | 0.0044 | rs75171718 | 0.0057 |
| 1 | *EXO1* | 4 | 242,011,492 | 242,053,241 | 0.0050 | rs1776178 | 0.0018 |
| 8 | *GSR* | 3 | 30,535,579 | 30,585,486 | 0.0066 | rs2161849 | 0.0069 |
| 6 | *GCLC* | 2 | 53,362,139 | 53,409,927 | 0.0077 | rs670548 | 0.0156 |
