# Supplementary material for: Sex‐ and APOE‐specific genetic risk factors for late‐onset Alzheimer's disease: Evidence from gene–gene interaction of longevity‐related loci
Source: Aging Cell. 2023 Aug 24;22(9):e13938. doi: 10.1111/acel.13938 (PMC10497850; doi:10.1111/acel.13938)
Supplement: Supplementary file 6 — Table S4. [file ACEL-22-e13938-s005.docx]

Table 3S: Results of eQTL Analysis of Top-SNPs from single-SNP (a) and gene-gene interactions (b) analysis

|  | **SNP** | **Assigned Gene** | **eQTL GTEx release V8** | **Regulome**  **Rank** | **Metabrain**  **Cortex (p-value)** | **Regulatory**  **Elements**  **SNPnexus** | **N_SNPs in LD**  **(r^2^ >=0.8)** | **Evidence of previous associations with age-related disease** |
| --- | --- | --- | --- | --- | --- | --- | --- | --- |
| a) | rs17810889 | C8orf49  (regulatory region, 1.8kb-3') | NEIL2  (Brain Hypothalamus)  RP11-481A20.10  (Brain Hypothalamus) | 1f | - | DNase1  H3K4me1 | 4  rs804269, rs7813191,  rs804267, rs60310672 | - |
|  | rs5742665 | IGF1 (intronic) | - | 1f | - |  | 0 | Increase in BMI in early life (Poole et al, 2012) |
|  | rs56190996 | IGF1R (intronic) | - | 5 |  | - | 0 | - |
|  | rs8113762 | IRGQ (3’ UTR) | XRCC1 (Thyroid) | 1f | XRCC1  (3.20e-16) | H3K4me1  H3K36me3 | 0 | - |
|  | rs3729587 | XPC (intronic) | XPC (Brain Cerebellum) | 1f | CHCHD4  (3.09e-8) | H3K4me1  H3K36me3 | 11  rs57663319, rs75616324, rs386584832, rs3731175,  rs60766604, rs3731149, rs73815319, rs73815317,  rs59306883, rs36208721, rs3731124 | Smoking and cancer risk (Liang et al, 2018) |
| b) | rs7563682 | AOX1 (intronic) | AOX3P (Skin) | 1f | - |  | 4  rs11684227, rs17467098,  rs60547830, rs201634428 | - |
|  | rs1776178 | EXO1 (5’ UTR) | EXO1 (Testis) | 1f | - | DNase1  H3K4me3 | 31  rs59504092, rs1635517, rs2526704, rs1635515, rs2236919, rs59245225, rs4149883, rs2488471, rs59783921, rs17233654, rs1776132, rs59653851, rs1776137, rs851780, rs1030763914, rs1776131, rs2488472, rs60036075, rs60620584, rs1635511, rs61296106, rs386540178, rs59790129, rs1627006, rs59281046, rs112640453, rs1635507, rs112909488, rs72632900, rs2526697, rs3819361, rs1635501 | - |
|  | rs2686186 | FDFT1 (5’ UTR) | NEIL2 (Adipose) | 1f | - |  | 4  rs2092005, rs9693470, rs2645440, rs10503424 | - |
|  | rs3757949 | GATA4 (intronic) | NEIL2 (Skin) | 1b | - |  | 0 | - |
|  | rs804281 | GATA4 (intronic) | OR7E161P (Brain Cortex),  RP11- 481A20.10  (Brain Cerebellum),  FAM167A (Brain Putamen),  FAM66A (Brain Caudate),  TDH (Brain Nucleus Accumbens) | 1f | FAM167A  (8.77e-20) | H3K27me3 | 1  rs56538482 | - |
|  | rs670548 | GCLC (intronic) | GCLC (Pituitary) | 1f | GCLC  (9.37e-17) | H3K4me3  H3K36me3 | 0 | - |
|  | rs7092522 | IDE (intronic) | EIF2S2P3 (Brain Substantia nigra; Brain Hypothalamus) | 1f | - |  | 20  rs6583822, rs6583824  rs2421941, rs9633673  rs7096101, rs17367314  rs1331989026, rs7894183  rs7914814, rs60949938  rs10882091, rs7071905  rs59117199, rs10736068  rs10736069, rs7900689  rs6583830, rs1589602364  rs143593173, rs9420589 | - |
|  | rs6214 | IGF1 (intronic) | - | 5 | - |  | 0 | Skeletal muscle mass  (Yang et al, 2015)  Obesity  (Grijalva-Avila et al, 2020)  Ischemic stroke  (Kim et al, 2012)  Cancer risk (Ong et a, 2014; Feik et al, 2010)  Non-alcoholic fatty liver disease (Sabzikarian et al, 2023) |
|  | rs1520220 | IGF1 (intronic) | WASHC3  (Esophagus-Mucosa) | 7 | - |  | 0 | Ischemic stroke (Kim et al, 2012); Coronary heart disease (Ricketts et al, 2011)  Cancer susceptibility (Wang et al, 2016; Xu et al, 2018) |
|  | rs1879612 | IGF1R (intronic) | - | 4 | - | H3K4me1 | 0 | - |
|  | rs12972985 | KLC3 (intronic) | KLC3  (Brain Caudate; Cortex; Nucleus Accumbens) | 1f | - | H3K27me3 | 6  rs62111442, rs61155201  rs12975413, rs12973593  rs1475355052, rs12977048 | - |
|  | rs62491484 | NEIL2 | NEIL2 (Brain Nucleus Accumbens; Putamen) | 1f | - |  | 0 | - |
|  | rs35435718 | PGPEP1L (intronic) | IGF1R (Brain Cerebellum) | 1f | PGPEP1L  (1.00e-23) |  | 0 | - |
|  | rs718630 | PTPN1 (regulatory region, 16 kb-5’) | PTPN1 (Brain Spinal cord) | 1f | - |  | 0 | - |
|  | rs28672744 | TXNRD1  (regulatory region) | EID3 (Adipose) | 1f | - | H3K36me3 | 0 | - |
|  | rs2010704 | XDH (intronic) | XDH (Skin) | 1f | - |  | 0 | - |
|  | rs4983559 | ZBTB42 | C14orf180 (Brain Caudate) | 1f | - |  | 6  rs11160819, rs11628510  rs45490496, rs2494747  rs2494748, rs2498786 | - |

The Table shows the most relevant results of eQTL analysis of associated SNPs.

NB: The symbol – is used when no significant result was found for the corresponding SNP. Investigated website were: <https://gtexportal.org/home/>; <https://regulomedb.org/regulome-search/> ; <https://www.metabrain.nl/cis-eqtls.html>. In GTEX, for those SNPs where Brain tissue was not available, the tissue with the highest p-value was reported. Regulome Rank scores indicate with a scale from 1 to 6 (the lowest is the value the more is significant) the likelihood for a variant to be located in a functional region, linked to the expression of a target gene and/or affecting the binding of a transcription factor. In MetaBrain (de Klein et al, 2023), significant (FDR<0.05) cis-eQTLs in Brain cortex are reported, with p-value referred to the top effect. In SNPnexus, regulatory elements are indicated according to RoadMap; tissue like brain and neurons are considered, and with H3K_me_ are indicated the epigenetic modifications to the DNA packaging protein Histone H3. For LD, the table indicates for each variant the SNPs showing a high to moderate LD (r^2^ >= 0.8). Data were retrieved by interrogating LDlink (<https://analysistools.cancer.gov/LDlink/?tab=home> ) and posing as coordinates the GRCh38 release, the Caucasian population, and default bp window.

**References**

Poole EM, Tworoger SS, Hankinson SE, Baer HJ. Genetic variability in IGF-1 and IGFBP-3 and body size in early life. BMC Public Health. 2012 Aug 15; 12:659. doi: 10.1186/1471-2458-12-659.

Grijalva-Avila J, Villanueva-Fierro I, Lares-Asseff I, Chairez-Hernández I, Rivera-Sanchez G, Martínez-Estrada S, Martínez-Rivera I, Quiñones LA, Loera-Castañeda V. Milk intake and IGF-1 rs6214 polymorphism as protective factors to obesity. Int J Food Sci Nutr. 2020 May;71(3):388-393. doi: 10.1080/09637486.2019.1666805.

Ong J, Salomon J, te Morsche RH, Roelofs HM, Witteman BJ, Dura P, Lacko M, Peters WH. Polymorphisms in the insulin-like growth factor axis are associated with gastrointestinal cancer. PLoS One. 2014 Mar 7;9(3):e90916. doi: 10.1371/journal.pone.0090916.

Yang CW, Li TC, Li CI, Liu CS, Lin CH, Lin WY, Lin CC. Insulin like Growth Factor-1 and Its Binding Protein-3 Polymorphisms Predict Circulating IGF-1 Level and Appendicular Skeletal Muscle Mass in Chinese Elderly. J Am Med Dir Assoc. 2015 May 1;16(5):365-70. doi: 10.1016/j.jamda.2014.11.015.

Feik E, Baierl A, Hieger B, Führlinger G, Pentz A, Stättner S, Weiss W, Pulgram T, Leeb G, Mach K, Micksche M, Gsur A. Association of IGF1 and IGFBP3 polymorphisms with colorectal polyps and colorectal cancer risk. Cancer Causes Control. 2010 Jan;21(1):91-7. doi: 10.1007/s10552-009-9438-4.

Kim HJ, Kim SK, Park HJ, Chung JH, Chun J, Yun DH, Kim YO. Polymorphisms of IGFI contribute to the development of ischemic stroke. Exp Ther Med. 2012 Jan;3(1):93-98. doi: 10.3892/etm.2011.372.

Sabzikarian M, Mahmoudi T, Tabaeian SP, Rezamand G, Asadi A, Farahani H, Nobakht H, Dabiri R, Mansour-Ghanaei F, Derakhshan F, Zali MR. The common variant of rs6214 in insulin like growth factor 1 (IGF1) gene: a potential protective factor for non-alcoholic fatty liver disease. Arch Physiol Biochem. 2023 Feb;129(1):10-15. doi: 10.1080/13813455.2020.1791187.

Wang Q, Liu L, Li H, Tao P, Qi Y, Li J. Effects of High-Order Interactions among IGFBP-3 Genetic Polymorphisms, Body Mass Index and Soy Isoflavone Intake on Breast Cancer Susceptibility. PLoS One. 2016 Sep 15;11(9):e0162970. doi: 10.1371/journal.pone.0162970.

Ricketts SL, Rensing KL, Holly JM, Chen L, Young EH, Luben R, Ashford S, Song K, Yuan X, Dehghan A, Wright BJ, Waterworth DM, Mooser V; GEMS Investigators; Waeber G, Vollenweider P, Epstein SE, Burnett MS, Devaney JM, Hakonarson HH, Rader DJ, Reilly MP, Danesh J, Thompson SG, Dunning AM, van Duijn CM, Samani NJ, McPherson R, Wareham NJ, Khaw KT, Boekholdt SM, Sandhu MS. Prospective study of insulin-like growth factor-I, insulin-like growth factor-binding protein 3, genetic variants in the IGF1 and IGFBP3 genes and risk of coronary artery disease. Int J Mol Epidemiol Genet. 2011 Aug 30;2(3):261-85.

de Klein N, Tsai EA, Vochteloo M, Baird D, Huang Y, Chen CY, van Dam S, Oelen R, Deelen P, Bakker OB, El Garwany O, Ouyang Z, Marshall EE, Zavodszky MI, van Rheenen W, Bakker MK, Veldink J, Gaunt TR, Runz H, Franke L, Westra HJ. Brain expression quantitative trait locus and network analysis reveals downstream effects and putative drivers for brain-related diseases, Nature Genetics, 2023, doi: https://doi.org/10.1038/s41588-023-01300-6
